# Asthma

**Published:** 1896-06-13

**Authors:** Patrick Watson Williams

**Affiliations:** Physician to Out-Patients and for Diseases of the Throat, Bristol Royal Infirmary


### ASTHMA.

By Patrick Watson Williams, M.D.Lond., Physician to Out-Patients and for Diseases of the Throat, Bristol Royal Infirmary.

For some years I have been specially interested in the disease in question, and I have latterly obtained far greater success in its treatment by throwing over almost entirely the time-honoured sedative remedies in favour of more tonic lines of treatment.

As the principles which will guide us in treatment will depend so largely on the particular pathological theories which appeal to us, I will refer to some of the moot points in the pathology of the disease which have recently been the subject of controversy, though the pathology and treatment of asthma, is, of course, far too large a subject to attempt, in the limited space at my disposal, to do more than present one or two points for special consideration.

In the present article I will consider, firstly, What is the dyspnœa due to? secondly, Why do paroxysms occur? thirdly, What therapeutic principles should guide us in seeking to cure the tendency to asthma and to relieve the attacks?

What is asthma? What are the most essential phenomena of asthma, the obstructive dyspnœa and the other well-known clinical manifestations of an attack, due to?

The older theory is that spasmodic contraction of the involuntary muscular fibres of the smaller bronchi occurs, causing partial stenosis and requiring increased inspiratory and expiratory efforts to make the air pass through the narrowed tubes. This is the view which still largely holds the field, but in 1872 the spasm theory was attacked by Weber, who contended that the asthmatic attack was due to a pares is of the pulmonary vaso-motor nerves, resulting in more or less sudden vaso-motor paralysis and consequently dilatation of the vessels in the bronchial mucous membrane, He believed that the vessels became engorged, and that by the turgescence of the mucous membrane the calibre of the bronchial tubes is markedly lessened and the passage of air through them is obstructed. This paralytic vascular engorgement, having lasted some hours, corresponding to the first or dry stage of the asthmatic paroxysm, exudation of serum and mucus ensues, with resulting relief of the engorgement, and cessation of the attack. Now it is well known that, as a rule, when the patient begins to expectorate watery mucus the attack is about to cease, and thus, at first sight, Weber's theory explains clinical observation. His theory has been extensively accepted, and a number of authorities have given up the bronchial spasm theory in favour of Weber's vaso-motor theory. Among his chief supporters are German Sée, and Bosworth, of New York. "Weber's paper was followed by a series of clinical observations, which largely lent weight to his theory, and also threw much light on the causes of the disease. The first observation of note was that of Voltolini, who reported a case of asthma due to the existence of nasal polypi, as shown by the fact that asthma promptly disappeared on the removal of nasal growths. This observation was followed by a number of similar reports, and gave rise to voluminous discussions, not only on asthma as a reflex disease due to nasal polypi, but also as due to other nasal disorders" (Bosworth). Turning now for a moment to hay-fever, we may recall to mind that this affection has been shown to be accompanied by vaso-motor dilatation in the nasal mucosa—in other words, it is a vaso-motor rhinitis, and while periodic hay-fever is often associated with hay-asthma, it is equally true that paroxysmal sneezing and running at the nose—*i.e.*. vaso-motor rhinitis—precedes, accompanies, or alternates with ordinary perennial asthma. I may go so far as to say that paroxysmal sneezing and running at the nose is absent in only a small percentage of asthmatics, vaso-motor rhinitis being one of the most constant phenomena of the asthmatic paroxysm, and must be regarded as part and parcel of the asthmatic syndrome. This is not fully recognised, for the reason that the asthmatic seldom complains of his own accord of the less troublesome nasal symptoms unless inquiry be made. It is very tempting, then, to follow Bosworth in his contention that the conditions of the mucous lining of the bronchioles are analogous to those observed in vaso-motor rhinitis, viz., vascular engorgement, followed by secretion of serum and sero-mucus.

But, on the other hand, I would urge that there is very good reason to believe that instead of the vessels of the mucous membrane of the bronchioles being dilated and engorged during at least the earlier stages of an asthmatic attack, they are markedly constricted, and the mucous membrane ischæmic. Firstly, I may point to the extreme pallor, cold extremities, the small, quick pulse, increased renal secretion, the confusion of thought or inability to think, and the inability to digest food which characterise the onset of a paroxysm as phenomena which are indicative of a wide-spread vascular constriction. Secondly, the asthmatic attacks are prone to occur under conditions which cause vaso-motor constriction, or increased arterial tension; for instance, emotion, fright, a cold wind, violent exercise, an undigested meal, &c. Thirdly, cardiac asthma occurs under similar conditions, and is associated with gout and the sclerotic kidney, which is equivalent to saying with vascular high tension. Fourthly, and I think this is conclusive evidence against the vascular-turgescence theory, the asthmatic paroxysm is cut short by the inhalation of amyl-nitrite, and is generally relieved by tri-nitrin, erythrol tetranitrate, iodide of potassium, a warm bath, and other vaso-dilators.

It may be urged that, while admitting the evidence of general vaso-motor constriction is conclusive, it is quite reasonable to maintain that the pulmonary artery, as well as the splanchnic area, is in a state of vaso-dilatation and vascular engorgement. To answer this objection I would refer to the careful and elaborate experiments of Bradford on the phenomena following excitation of the vaso-motor centre recorded in the Journal of Physiology. He shows that excitation of the centre produces a rise of blood pressure in the pulmonary artery by causing an active constriction of the lung vessels (the vagi being divided), the vaso-constricting fibres not running in the vagi, but leaving the cord by the upper dorsal nerves. But it is more important still to bear in mind that the bronchial mucous membrane is supplied by systemic arteries, not from the pulmonary artery, but from the aorta, and they at least are involved in the general vaso-motor constriction. There can scarcely be any need for me to adduce evidence that asthma is largely dependent on irritation of the vagus acting reflexly on the vagus centre, and I may remark that Bradford found that excitation of the central end of a divided vagus induced rise of blood pressure in the pulmonary arteries, and moreover, that this was followed by reactionary depression after shutting off the current. Do not his results corroborate the theory that during the early stage of asthma there is bronchiole and pulmonary ischæmia, followed by bronchial congestion and exudation?

When I first began to use amyl-nitrite in asthmatics I thought that the fact that it amost invariably cut short the attack whilst its very transient effect lasted, proved that the asthmatic paroxysm was due to vaso-motor constriction alone, but one must remember that it is one of the most potent remedies we possess for relieving muscular spasm, that it will at once relieve spasmodic croup and many other conditions due to muscular spasm. It is very interesting, however, to note that while relieving the dyspnœa of asthma, it will relieve also the obscurity of thought, which is sometimes a notable symptom in asthma, thus suggesting that the pronounced vaso-constriction extends to the brain cortex. Furthermore, I may point to the relief afforded by the inhalation of chloroform, morphine, &c., which do not cause vascular turgescence. Therefore, while it is possible that the dyspnœa is in some measure due to pulmonary ischæmia, we must abandon the theory of vascular turgescence as the cause of the mechanical obstruction to the passage of air in the bronchioles, and also abandon the idea that vaso-motor constriction is the sole cause of dyspnœa.

What direct evidence have we that broncho-spasm occurs? Let us turn to Lazarus' experiments. He determined the resistance which the passage of air to and from the alveoli encountered during artificial respiration. The vagus was divided in curarized animals rendered apnœic, and the peripheral end of the vagus stimulated by a very feeble electrical current. This caused an increase in the resistance to the passage of air in the bronchioles. But, as owing to the division of the vagus, the pulse increased in frequency, the question arose as to whether the contraction of the bronchial muscles or changes in the circulatory system, more especially in the vessels of the bronchial mucous membrane, caused this increased resistance. Therefore the vaso-motor influence of the vagus was eliminated by the injection of atropine. Yet the same increased resistance was obtained as before, proving that it was due to bronchial spasm. Einthoven obtained similar results when he eliminated blood pressure from vaso-constriction by first bleeding the animal to death. Another point of interest in Lazarus' experiments was the result of stimulation of the nasal mucous membrane, viz., a further increase in the intra-bronchial pressure. But this increase in intra-bronchial pressure did not occur on stimulating the nose after division of the vagus, showing that it is through the vagus that the bronchial spasm was induced by irritation in the nose. Bischoff has observed asthma follow nasal cauterisation. This is certainly an almost unique experience, for, though dyspnœa has followed intra-nasal irritation in the lower animals, asthma has not been artificially induced by nasal irritation in healthy human subjects. Nevertheless it proves the possible dependence of bronchial spasm on nasal excitation, and of this we have very ample clinical corroboration. Not that I suggest for a moment that the nose is the invariable peripheral source of reflex bronchial spasm. We know that irritation of the vagus in stomach, heart, intestines, &c., must be held to account for the attacks in many cases.

I think there is sufficient evidence to warrant our assuming that the asthmatic attack is attended with both spasm of the bronchioles and constriction of the vessels of the bronchial mucous membrane, and that together with the relaxation of the bronchial spasm, there is a reactionary vaso-motor paresis, and more or less bronchorrhœa. Sometimes this bronchorrhœa closely simulates true bronchitis, or the vascular congestion may originate a form of bronchitis, so that asthma may perhaps be found to cause bronchitis and a liability to attacks of true bronchitis, quite as often as bronchitis causes asthma.

I read with great interest the very original article on the pathology of bronchitis by Professor Thomson,[1] of the New York University. He draws attention to the significant fact that an inflammation beginning in the nasal cavity constantly traverses the crossing of the two tracts of respiration and deglutition in the pharynx on its way to the larynx and bronchi, while it scarcely ever turns into the mouth or down the œsophagus. The mucous membranes do not became inflamed simply because they are mucous membranes, for in this case not even the closest proximity suffices to involve one of the two in an affection of the other. This points strongly to a specific nervous interconnection between the upper and lower respiratory passages which follows the law of vaso-motor association.

Why do the paroxysms occur?

It is very difficult to answer this question until we know more definitely the physiological action of the bronchial involuntary muscular tissue. It is out of the question to presume that it has no physiological purport, and it would seem that just as the arterioles, in contradistinction to the large arteries, dilate or contract so as to regulate the blood pressure beyond, so the bronchioles, in contradistinction to the trachea and bronchi, dilate or contract so as to regulate, according to the needs of the economy or a varying environment, the ingress or egress of air to the alveoli of the lungs. Just as it is in the untranquil conditions we observe most readily the extreme degrees of vaso-motor constriction or dilation, so do we observe the rhythmical dilatation and contraction of the nostrils and of the glottic opening, in inspiration and expiration, to be most marked in conditions of dyspnœa or under emotion; and so may we not also assume that the bronchioles which we cannot inspect dilate and contract, and moreover, maintain a condition of more or less persistent high or low bronchiole tension, just as our vaso-motor apparatus maintains a more or less persistent high or low arterial tension?

[1] N.Y. Med. Journ., May 16, 1896.

